# An integrative model of multi-organ drug-induced toxicity prediction using gene-expression data

**DOI:** 10.1186/1471-2105-15-S16-S2

**Published:** 2014-12-08

**Authors:** Jinwoo Kim, Miyoung Shin

**Affiliations:** 1 Bio-Intelligence & Data Mining Lab, School of Electronics Engineering, Kyungpook National University, Daegu, Korea

## Abstract

**Background:**

In practice, some drugs produce a number of negative biological effects that can mitigate their effectiveness as a remedy. To address this issue, several studies have been performed for the prediction of drug-induced toxicity from gene-expression data, and a significant amount of work has been done on predicting limited drug-induced symptoms or single-organ toxicity. Since drugs often lead to some injuries in several organs like liver or kidney, however, it would be very useful to forecast the drug-induced injuries for multiple organs. Therefore, in this work, our aim was to develop a multi-organ toxicity prediction model using an integrative model of gene-expression data.

**Results:**

To train our integrative model, we used 3708 *in-vivo *samples of gene-expression profiles exposed to one of 41 drugs related to 21 distinct physiological changes divided between liver and kidney (liver 11, kidney 10). Specifically, we used the gene-expression profiles to learn an ensemble classifier for each of 21 pathology prediction models. Subsequently, these classifiers were combined with weights to generate an integrative model for each pathological finding. The integrative model outputs the likeliness of presenting the trained pathology in a given test sample of gene-expression profile, called an *integrative prediction score *(IPS). For the evaluation of an integrative model, we estimated the prediction performance with the *k*-fold cross-validation. Our results demonstrate that the proposed integrative model is superior to individual pathology prediction models in predicting multi-organ drug-induced toxicities over all the targeted pathological findings. On average, the AUC of the integrative models was 88% while the AUC of individual pathology prediction models was 68%.

**Conclusions:**

Not only does this integrative model produce comparable prediction performance to existing approaches, but also it produces very stable performance overall. In addition, our approach is easily expandable to a variety of other multi-organ toxicology applications.

## Background

With the advent of high-throughput technologies (such as microarrays), an explosive growth of gene-expression data in toxicology applications has given us new insights into the biological processes of hepatotoxicity or nephrotoxicity [1]. Thus, many studies have worked on using gene-expression profiles for the prediction of the potential negative effects of new drugs under development [1,2]. Because new drugs often lead to some injuries in the liver or kidney, which present various pathological findings, it would be very useful to predict the drug-induced toxicity for multiple organs. However, thus far, most of the earlier works focused on predicting single-organ toxicity with only limited pathological symptoms [3-9].

For example, Cui et al. [3] used gene-expression profiles obtained from kidney RNA samples with rat cDNA microarrays to develop an SVM-based prediction model that groups gene-expression profiles into four classes according to the severity and type of pathology. Using this model, they could predict the pathologies of 28 test profiles with 100% specificity and 82% sensitivity. Lingkang et al. [4] grouped liver samples by the level of necrosis exhibited in the tissue and used them to develop a random forest classifier with 21 genes. They achieved 90%, 80%, and 60% prediction accuracies against test data derived from the livers of rats exposed to three different hepatotoxins, respectively. Kedar et al. [5] proposed a network model to assess chronic hepatotoxicity based on sub-chronic hepatic gene-expression data in rats, where the directed graph accounts for the interactions among drugs, differentially expressed genes and chronic hepatotoxicity. They estimated phenotypical exposure risk such as toxic hepatopathy, diffuse fatty change and hepatocellular adenoma from gene-expression profile of rats. Low et al. [6] proposed a hybrid model of chemical descriptors and gene-expression data to predict hepatotoxicity. Although the hybrid models did not show better accuracy than the model based on only gene-expression data, the use of chemical descriptors could enrich the interpretation of the model. Minowa et al. [7] developed the prediction model for the onset of drug-induced proximal tubular injury from gene-expression data, and achieved 93% sensitivity and 90% specificity. Bowles et al. [8] quantified several pathological findings and DILI severity and then constructed statistical prediction models from gene-expression profiles for liver pathology in rats and for drug-induced liver injury. For this purpose, they used the Lasso regression and "glmnet" algorithm to extract models for rat liver pathology prediction and used stochastic gradient boosting to extract models for drug-induced liver injury. Zhang et al. [9] employed gene-expression data, cell-based assays, and pathological data to obtain a network of early-response genes as a consensus signature of drug-induced *in-vitro *and *in-vivo *toxicity, and used the network to predict liver or kidney toxicity from gene-expression data. The accuracy of prediction model was between 80% and 97% in both liver and kidney.

In this work, we attempt to develop an integrative model of drug-induced toxicity prediction applicable to the prediction of multiple organ pathologies. To achieve this end, we investigated the relationships between distinct pathologies by exploring co-occurrences of pathologies within training samples, and used them to combine individual pathology prediction models for drug-induced toxicity. Consequently, for a test sample, our integrative model can predict the occurrence of the trained pathologies presented in multiple organs.

## Methods

### Target pathological findings in multi-organs

For experiments, our toxicity prediction model targets 11 liver physiological changes (pathological findings) that signal the presence of developing pathology (Eosinophilic change, Cellular infiltration, Hypertrophy, Degeneration, Pigment deposit, Increased mitosis, Necrosis, Single cell necrosis, Cytoplasmic vacuolization, Proliferation, Fibrosis) and 10 kidney pathological findings (Cellular infiltration, Hypertrophy, Degeneration, Necrosis, Hyaline droplet, Hyaline cast, Dilatation, Basophilic change, Cytoplasmic vacuolization, Regeneration), carefully chosen from Open TG-GATEs http://toxico.nibio.go.jp[10].

In fact, according to TG-GATEs, the researchers extracted the slides of the liver or kidney from all in-vivo samples, and pathologists examined these slides to detect pathological symptoms and their severity in various parts of the liver or kidney. (See Additional file 1, 2) Due to the limitation of available gene-expression data in both the liver and kidney, which cannot cover all possible pathological symptoms, we targeted only the 21 findings, as mentioned above.

For 37 liver and 26 kidney drug-induced pathological findings observed in *in-vivo *rat samples exposed to one of 131 drugs in TG-GATEs, we simplified them into general terms (e.g., grouping the subtypes of liver degeneration into '*liver degeneration*', or grouping the necrosis of bile duct or hepatocyte into *liver necrosis*.) and then selected only the pathology terms that were induced by at least 5 drugs or more, as the targets of our prediction model.

### Training data for toxicity prediction model

For the learning of the toxicity prediction model, we used confirmed case and control samples, with gene-expression profiles for cases with targeted pathological findings and expression profiles for controls without the targeted pathological finding, respectively. All the gene-expression profiles in the training data were normalized by using a robust multi-array average (RMA) method with the RefPlus R package [11], where the reference model was built by selecting 5% of the training samples in each organ and used to derive reference quartiles and probe effects for normalization. To construct the toxicity prediction model, we need to identify gene signatures particular to each of the 21 pathological findings. For this purpose, we applied the *t*-test to the training data of confirmed case/control samples in each pathological finding and selected the top 5% ranked genes in the increasing order of p-values.

### Construction of individual pathology prediction models

For each of the 21 pathological findings, we trained an ensemble model of *n k*-nearest neighbor (KNN) classifiers (i.e., an ensemble of *n *sub-prediction models) to construct an individual pathology prediction model. The main reason of developing *n *sub-prediction models for each pathology prediction is to handle the data imbalance problem (i.e., handling the situation in which the number of case samples is much smaller than the number of control samples), a problem that commonly occurs in training data for adverse drugs reaction [12,13]. Each sub-prediction model was developed with each of *n *jackknife samples generated from training data, in which each jackknife sample includes all the confirmed case samples and a randomly selected subset of control samples. The final output of any individual pathology prediction model is determined by aggregating all the outputs of *n *sub-prediction models for each pathology finding. That is, for a test sample *s *of gene-expression profile with exposure to a certain drug, our pathology prediction model produces the *pathology prediction score *(PPS)$M_{F_{i}}\left(s\right)$ as an output for a pathology finding *F_i _*by averaging all the outputs of *n *sub-prediction models as follows:

$$M_{F_{i}}\left(s\right)=\frac{1}{n}\sum_{k=1}^{n}sub\text{\_}M_{F_{i}}^{k}\left(s\right)$$

**Figure 1:**
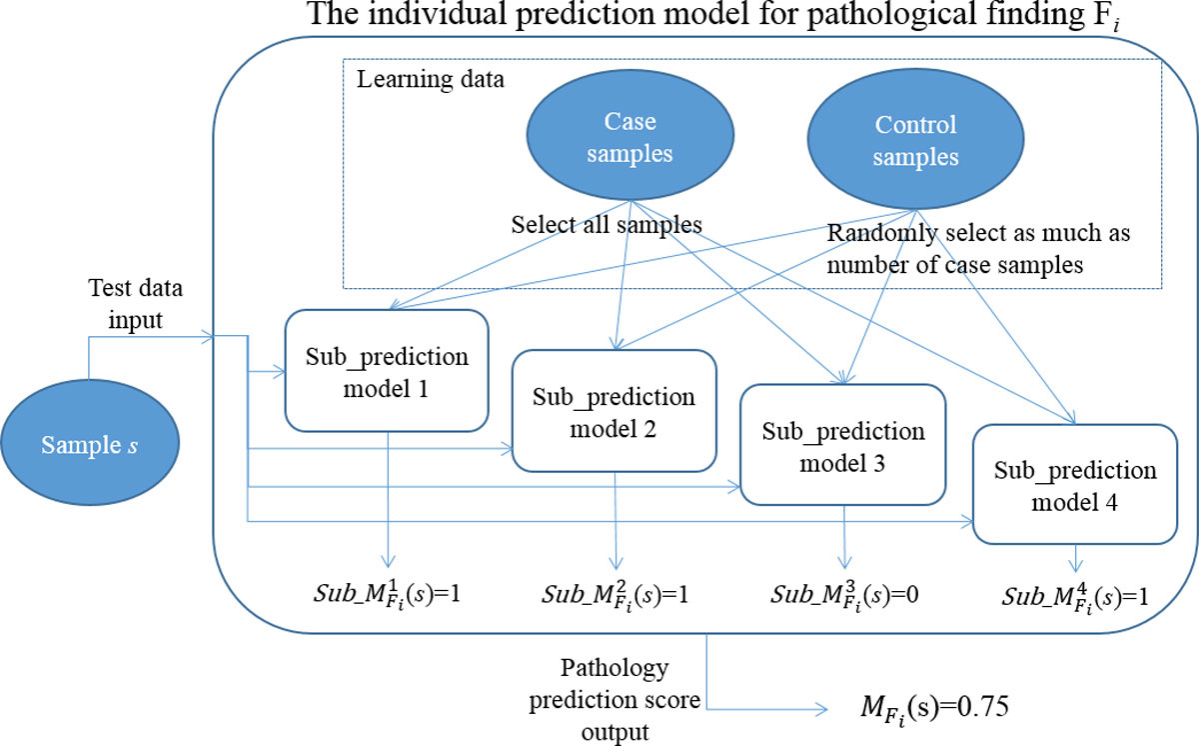
**The procedure of producing the pathology prediction score (PPS) in an individual pathology prediction model**.

The score of M*_F_*(*s*) is in the range of 0[1], where values approaching 0 indicate that a pathological finding F will not occur in the sample *s *and values closer to 1 indicate that F will occur in the sample *s*. If M*_F_*(*s*) is higher than a certain threshold, we predict that the pathological finding F would occur in the sample *s*. Figure 1 illustrates the procedure of producing the PPS as an output of individual pathology prediction.

### Pathology relationships extraction

To investigate the relationships between pathological findings for drug-induced toxicity prediction, we constructed a *pathology occurrence matrix *that describes the presentation states of training samples for the targeted pathology findings (i.e., 11 liver and 10 kidney). From this matrix, we can derive the *pathology occurrence vector *for each pathology finding and use them to calculate the Jaccard similarities between confirmed case samples displaying two different pathological symptoms. Specifically, assuming that there are *n *training samples and *k *pathological findings of interest, the pathology occurrence matrix Φ is defined by:

$$\text{Φ}=\left[\begin{array}{ccc} {\text{Φ}}_{1} & \cdots \; & {\text{Φ}}_{k} \end{array}\right]=\left[\begin{array}{ccc} \varphi_{11} & \cdots \; & \varphi_{1k}\\ \vdots & \ddots & \vdots \\ \varphi_{n1} & \cdots \; & \varphi_{nk} \end{array}\right]$$

Where φ*_ij _*= if a pathology F*_j _*occurred in the *i*^th ^training sample and φ*_ij _*otherwise. Also, the similarity between F*_i _*and F*_j _*is defined as the Jaccard coefficient between their pathology occurrence vectors (i.e., Φ*_i _*and Φ*_j_*) which measures the commonness of sample occurrences of two pathology findings. Formally, the pathology similarity S between F*_i _*and F*_j _*is calculated by:

$$\text{S}\left(F_{i},F_{j}\right)=Jaccard\left({\text{Φ}}_{i},{\text{Φ}}_{j}\right)=\frac{N_{11}}{N_{01}+N_{10}+N_{11}}$$

where *N*_01 _is the total number of training samples in which F*_i _*does not occur and F*_j _*does occur, *N*_10 _is the total number of training samples in which F*_i _*occur and F*_j _*does not occur, and *N*_11 _is the total number of training samples in which both F*_i _*and F*_j _*does occur. The value of pathology similarity is in the range of 0[1], and some examples of pathology similarities for 4 pathological findings (i.e., liver eosinophilic change, liver cellular infiltration, kidney cytoplasmic vacuolization, and kidney regeneration) are shown in Table 1.

**Table 1:** Examples of the pathology similarities for 4 pathological findings.

	Liver Change, eosinophilic	Liver Cellular infiltration	Kidney Vacuolization, cytoplasmic	Kidney Regeneration
**Liver Change, eosinophilic**	1.00	0.21	0.05	0.00

**Liver Cellular infiltration**	0.21	1.00	0.02	0.04

**Liver Hypertrophy**	0.04	0.10	0.08	0.12

**Liver Degeneration**	0.00	0.20	0.00	0.10

**Liver Deposit, pigment**	0.02	0.05	0.00	0.02

**Liver Increased mitosis**	0.08	0.04	0.00	0.02

**Liver Necrosis**	0.05	0.32	0.03	0.05

**Liver Single cell necrosis**	0.04	0.20	0.00	0.06

**Liver Vacuolization, cytoplasmic**	0.05	0.00	0.14	0.08

**Liver Proliferation**	0.04	0.14	0.08	0.07

**Liver Fibrosis**	0.00	0.15	0.05	0.04

**Kidney Cellular infiltration**	0.00	0.01	0.00	0.11

**Kidney Hypertrophy**	0.00	0.02	0.00	0.04

**Kidney Degeneration**	0.04	0.04	0.09	0.29

**Kidney Necrosis**	0.10	0.02	0.11	0.16

**Kidney Hyaline droplet**	0.01	0.00	0.05	0.10

**Kidney Cast, hyaline**	0.02	0.02	0.19	0.11

**Kidney Dilatation**	0.04	0.01	0.05	0.16

**Kidney Change, basophilic**	0.02	0.02	0.03	0.00

**Kidney Vacuolization, cytoplasmic**	0.05	0.02	1.00	0.03

**Kidney Regeneration**	0.00	0.04	0.03	1.00

The rationale of pathology relationships can be explained as follows: A drug-induced toxicity can present several pathological findings in liver or kidney, and the occurrence of certain pathological finding may be associated with the occurrence of some other pathological finding(s). This is because a drug-induced toxicity can perturb certain gene-expressions or protein activities followed by the perturbation of several pathway activities, leading to various toxicity symptoms [14]. For example, the PPAR-alpha gene is related to liver hypertrophy [15], which is also related to kidney tubular necrosis [16,17]. ARG1 gene is related to both biliary injury and single cell necrosis [18]. Hence, some symptom(s) of toxicity might be the indirect evidence of other symptom(s) of toxicity. To implement this rationale in our experiments, we estimated the *degree *of the association between two pathological findings by calculating the Jaccard similarity between their pathology occurrence vectors.

### The integrative model for drug-induced toxicity prediction

The construction of the integrative model for drug-induced toxicity prediction is done by the weighted combination of all the 21 individual pathology prediction models. For this purpose, we first obtain the normalized pathology similarities N(*F_i_, F_j_*) from the pairwise pathology relationships S(*F_i_, F_j_*) defined as above by exploring co-occurrence samples of two pathological findings. That is, when having *k *different pathological findings, the normalized pathology similarity between F*_i _*and F*_j _*is defined by

$$\text{N}\left(F_{i},F_{j}\right)=\frac{\text{S}\left(F_{i},F_{j}\right)}{\sqrt{\sum_{m=1}^{k}\text{S}{\left(F_{i},F_{m}\right)}^{2}}}$$

Then, our integrative model produces the *integrative prediction score $\text{IP}{\text{S}}_{F_{i}}\left(s\right)$*of a test sample *s *for each pathological finding F*_i_*, which is calculated by

$$\text{IP}{\text{S}}_{F_{i}}\left(\text{s}\right)=\sum_{m=1}^{k}\text{N}\left(F_{i},F_{m}\right)\cdot {\text{M}}_{F_{m}}\left(s\right)$$

If the score of $\text{IP}{\text{S}}_{F_{i}}\left(s\right)$ is higher than a certain threshold, it is predicted that the pathology finding F*_i _*would occur in the test sample *s*. Thus, for *k *distinct pathological findings, our integrative model produces a *k*-dimensional vector of the IPSs of a test sample *s *as follows:

$$IPS\left(s\right)=\left[\begin{array}{c} \text{IP}{\text{S}}_{F_{1}}\left(s\right)\\ \text{IP}{\text{S}}_{F_{2}}\left(s\right)\\ \text{IP}{\text{S}}_{F_{3}}\left(s\right)\\ \vdots \\ \text{IP}{\text{S}}_{F_{k}}\left(s\right) \end{array}\right]=\left[\begin{array}{ccccc} \text{N}\left(F_{1},F_{1}\right) & \text{N}\left(F_{1},F_{2}\right) & \text{N}\left(F_{1},F_{3}\right) & \ldots & \text{N}\left(F_{1},F_{k}\right)\\ \text{N}\left(F_{2},F_{1}\right) & \text{N}\left(F_{2},F_{2}\right) & \text{N}\left(F_{2},F_{3}\right) & \ldots & \text{N}\left(F_{2},F_{k}\right)\\ \text{N}\left(F_{3},F_{1}\right) & \text{N}\left(F_{3},F_{2}\right) & \text{N}\left(F_{3},F_{3}\right) & \ldots & \text{N}\left(F_{3},F_{k}\right)\\ \vdots & \vdots & \vdots & \ddots & \vdots \\ \text{N}\left(F_{k},F_{1}\right) & \text{N}\left(F_{k},F_{2}\right) & \text{N}\left(F_{k},F_{3}\right) & \ldots & \text{N}\left(F_{k},F_{k}\right) \end{array}\right]\cdot \left[\begin{array}{c} {\text{M}}_{F_{1}}\left(s\right)\\ {\text{M}}_{F_{2}}\left(s\right)\\ {\text{M}}_{F_{3}}\left(s\right)\\ \vdots \\ {\text{M}}_{F_{k}}\left(s\right) \end{array}\right]$$

### An illustrative example of the integrative model

**Figure 2:**
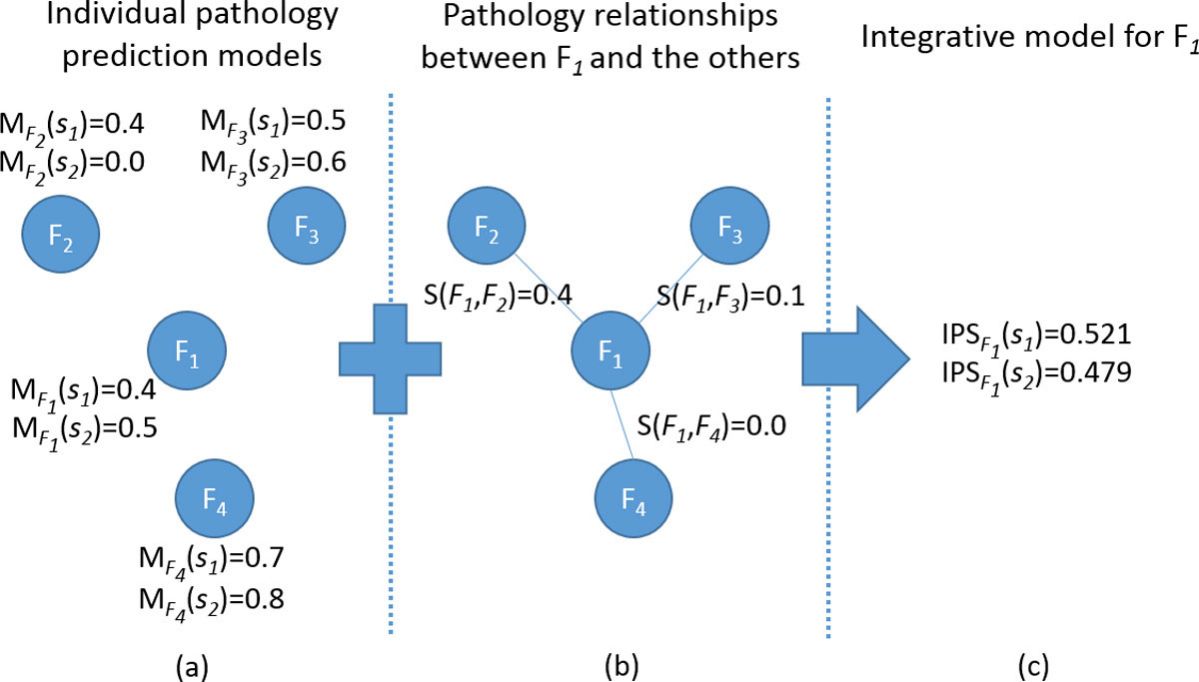
**An illustrative example of integrative model**.

For better understanding, we illustrate the integrative model by a toy example (Figure 2). Suppose that we have 4 different pathological findings of interest. Firstly, we develop 4 individual pathology prediction models, producing $M_{F_{i}}\left(s\right)$, *i *= 1, ..., 4, as in Figure 2(a). Secondly, the relationships among the 4 pathological findings are extracted by exploring co-occurrence cases of two different findings in training data, as in Figure 2(b). Thus, if we assume that $M_{F_{1}}\left(s_{1}\right)=0.4$, $M_{F_{2}}\left(s_{1}\right)=0.4$, $M_{F_{3}}\left(s_{1}\right)=0.5$, and $M_{F_{4}}\left(s_{1}\right)=0.7$ for a sample s*_1 _*and $M_{F_{1}}\left(s_{2}\right)=0.5$, $M_{F_{2}}\left(s_{2}\right)=0.0$, $M_{F_{3}}\left(s_{2}\right)=0.6$, and $M_{F_{4}}\left(s_{2}\right)=0.8$ for a sample s*_2_*, our integrative model would produce the scores of $\text{IP}{\text{S}}_{F_{1}}\left(s_{1}\right)=0.521$ and $\text{IP}{\text{S}}_{F_{1}}\left(s_{2}\right)=0.479$, as shown in Figure 2(c). From this example, we can notice that the IPS for a certain pathological finding is affected by both the degree of the association with other pathology findings and the results of other pathology prediction models. That is, in the case of a pathological finding F*_1 _*of s*_1_*, the score of $\text{IP}{\text{S}}_{F_{1}}\left(s_{1}\right)=0.521$ gets higher than the score $M_{F_{1}}\left(s_{1}\right)=0.4$ of an individual pathology prediction model because its highly associated finding F_2 _produces a relatively high score of $M_{F_{2}}\left(s_{1}\right)=0.4$. On the other hand, in the sample of s*_2_*, the $\text{IP}{\text{S}}_{F_{1}}\left(s_{2}\right)=0.479$ gets lower than the score $M_{F_{1}}\left(s_{2}\right)=0.5$ of an individual pathology prediction model because its highly associated finding F*_2 _*produces a low score of $M_{F_{2}}\left(s_{2}\right)=0.0$. Thus, if the threshold of IPS is set to 0.5, our model predicts that the pathological finding F*_1 _*will be presented in the sample s*_1 _*and not in the sample s*_2_*.

## Results and discussion

### Evaluation of individual pathology prediction models for multi-organ drug-induced toxicity

To evaluate the performance of individual pathology prediction models in forecasting multi-organ drug-induced toxicity over the targeted pathological findings, we used 3708 rat *in-vivo *samples exposed to one of our chosen 41 drugs. (See Additional file 3) Here, 41 drugs were chosen from TG-GATEs of which the gene expression profiles are provided in both the liver and kidney samples in all cases. As discussed above, we developed 21 individual pathology prediction models for our targeted pathological findings (i.e., 11 in liver and 10 in kidney) using the KNN-based ensemble classifiers, which produced the outputs of the pathology prediction scores (PPSs). If the PPS output of a pathology prediction model for a test sample is greater than a given threshold, then we predict that the targeted pathological finding will occur in the test sample.

We estimated the prediction performance of each individual pathology prediction model in terms of sensitivity and specificity using the *k*-fold cross-validation method. That is, original training data of the *in-vivo *samples mentioned in Methods were divided into *k *groups of equal size in the unit of drugs. To avoid an over-fitting problem, we used the samples of *k-*1 groups for model development (including gene signature selection for the targeted pathology, pathology relationship extraction, and prediction model learning), and used the samples of 1 remaining group to test model performance. This process was iterated *k *times for different selection of a test group (See Additional file 4). Here, the threshold value of PPS in each pathology prediction model was set to maximize the geometric mean of sensitivity and specificity. The geometric mean of sensitivity and specificity is often used to evaluate a prediction model with imbalanced data set.

**Figure 3:**
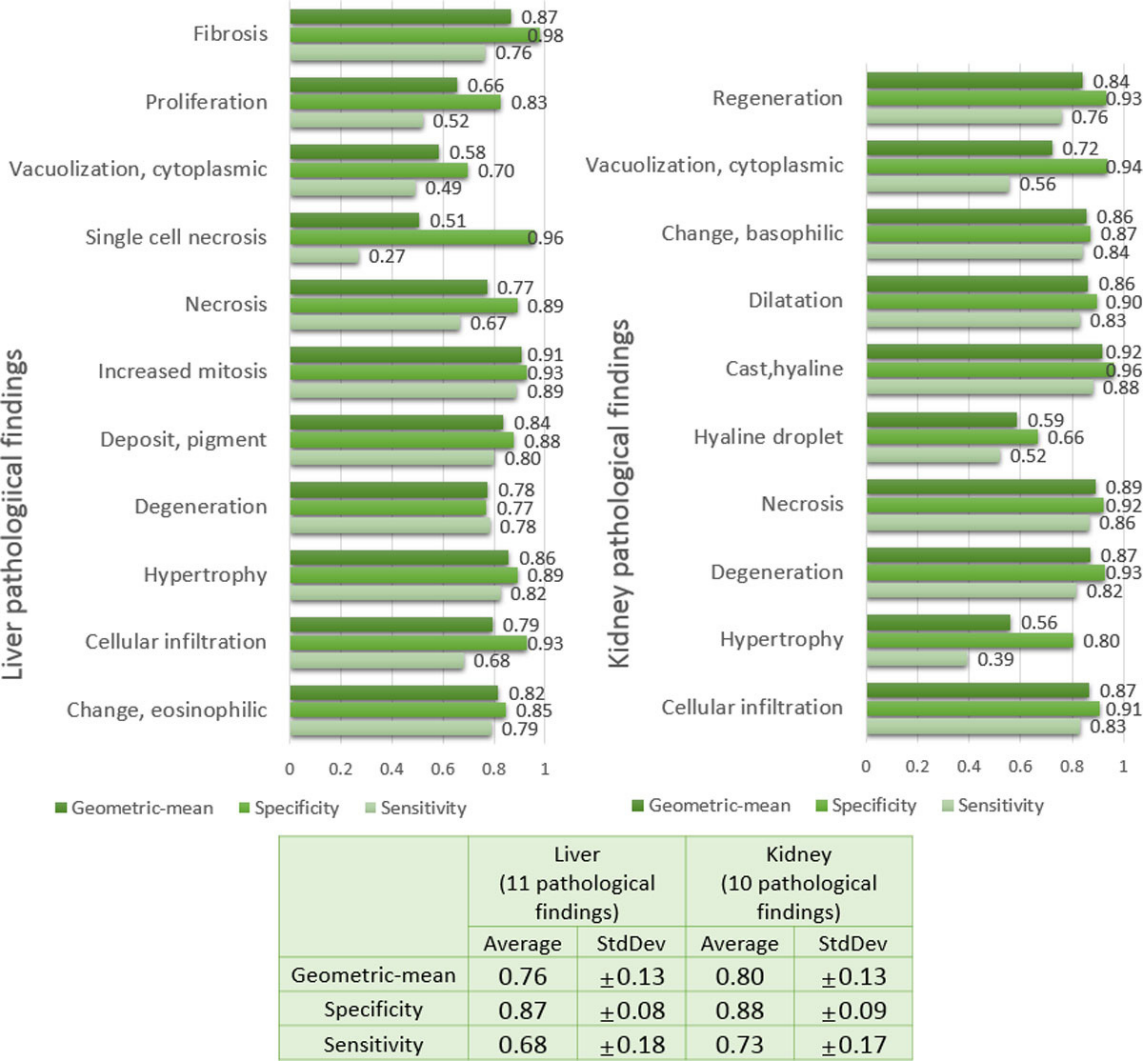
**Evaluation of the individual prediction models in forecasting 11 liver and 10 kidney pathological findings**.

**Figure 4:**
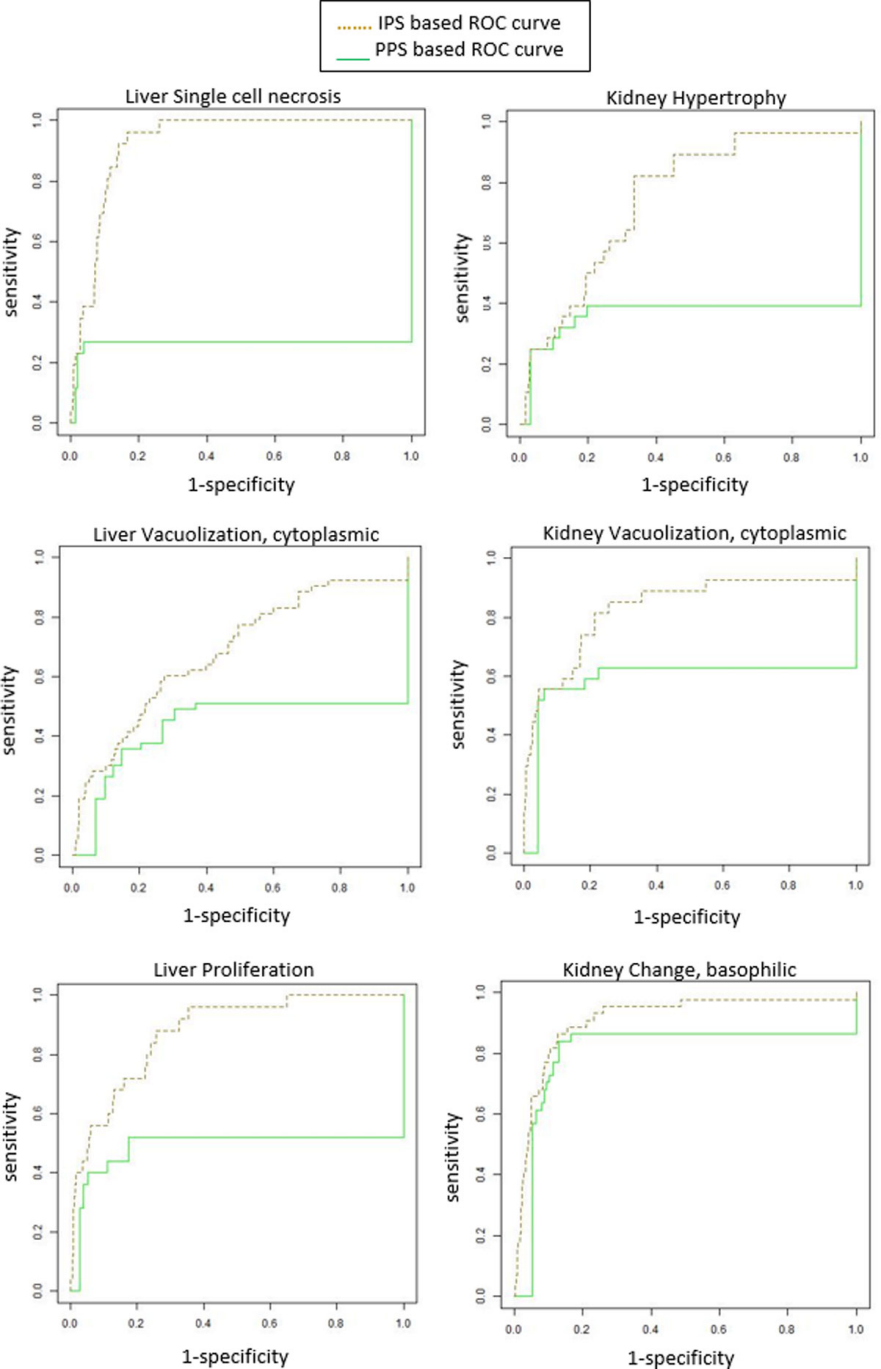
**ROC curves of the pathology prediction models regarding 3 liver and 3 kidney pathological findings**.

Figure 3 displays the evaluation results of the 21 individual pathology prediction models. As seen in this figure, all the individual pathology prediction models tend to show relatively lower sensitivities (approximately 70% on average) than specificities (approximately 87% on average) overall in predicting the targeted pathological findings. In addition, the standard deviations of sensitivity over all the models seems to be considerably high, ±18% in liver pathology and ±17% in kidney pathology, compared to those of specificity, i.e., ±8% in liver pathology and ±9% in kidney pathology. Even the sensitivities of several pathology prediction models (e.g., models for liver single cell necrosis and kidney hypertrophy) are extremely low as <50%. Since the performance of individual pathology prediction models depends on the choice of the PPS thresholds, it is worthwhile to draw ROC curves for model evaluation. The ROC curves of 6 pathology prediction models, which show the most distinction between PPS-based models and IPS-based models, are given in Figure 4. All the ROC curves of 21 pathology prediction models are also given in Additional file 5.

### Evaluation of the proposed integrative models for multi-organ drug-induced toxicity

We evaluated the prediction performance of the proposed integrative models in the same manner as for the individual pathology prediction models, using the *k*-fold cross-validation method. Particularly, we extracted pathology relationships only based on the samples of *k*-1 groups chosen as training set. (See Additional file 4) Here, the threshold of IPS was also set to maximize the geometric mean of sensitivity and specificity in forecasting the targeted pathological changes.

**Figure 5:**
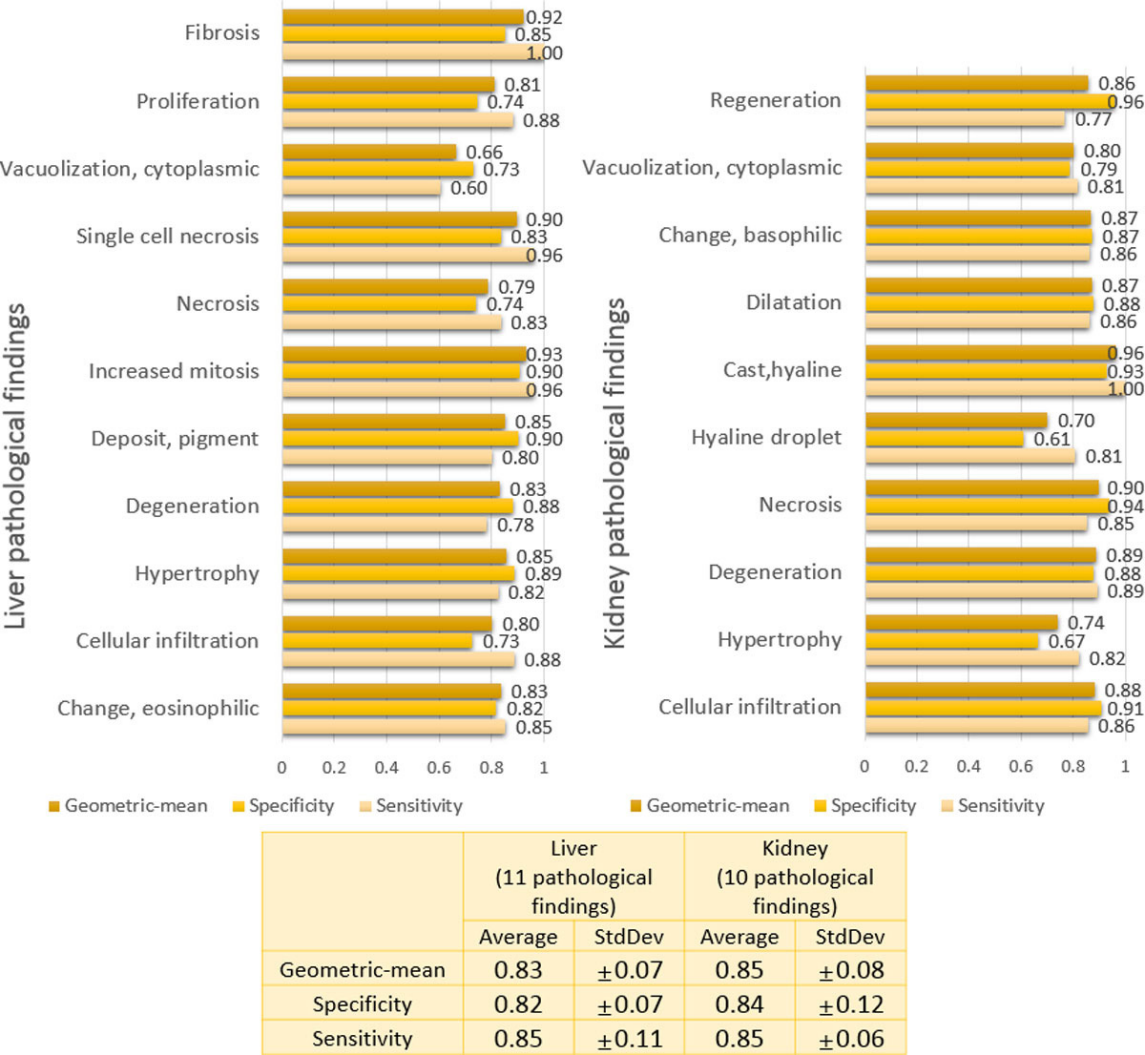
**Evaluation of the proposed integrative models in forecasting 11 liver and 10 kidney pathological findings**.

**Figure 6:**
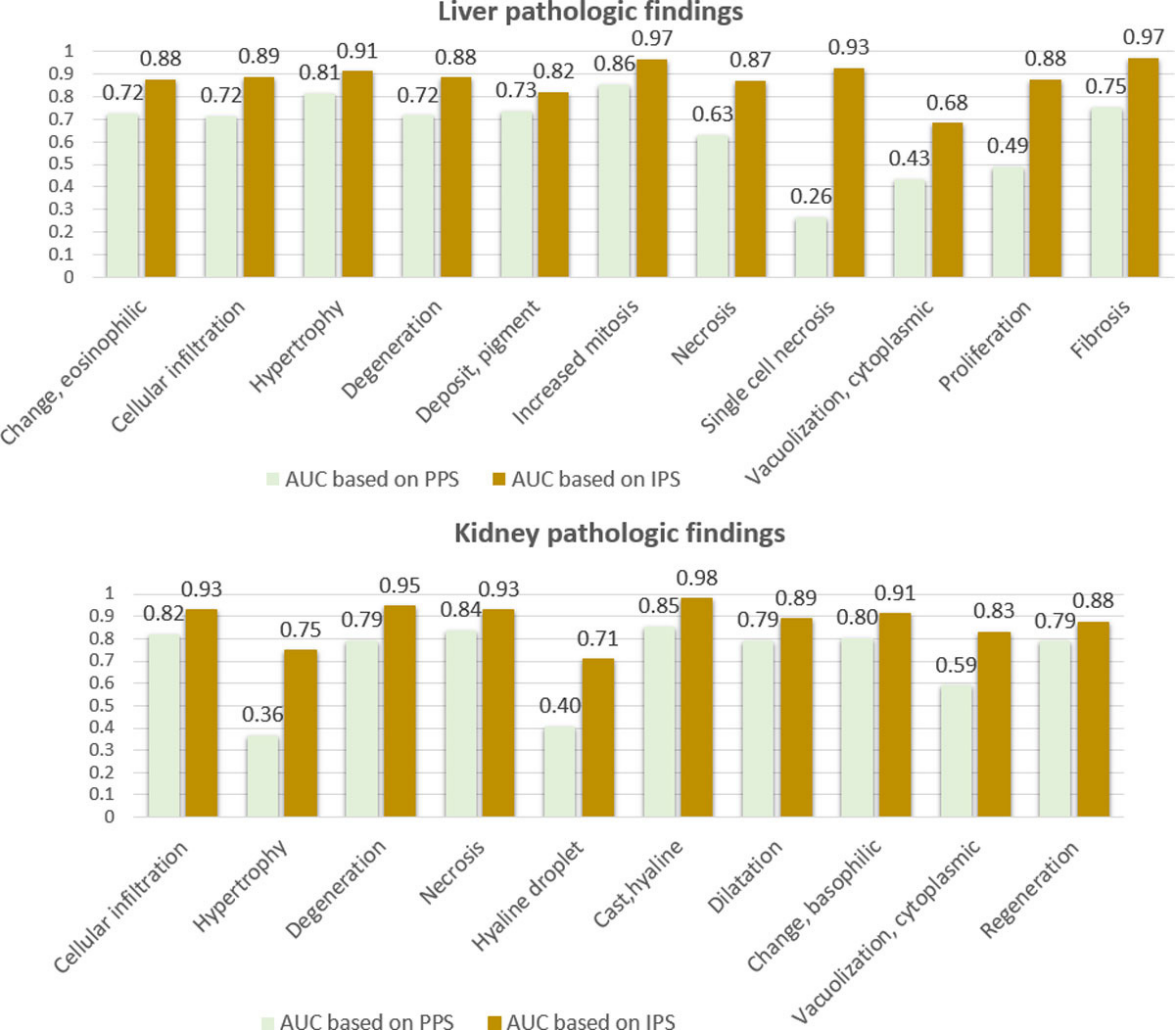
**Comparison of prediction performance in the IPS-based integrative models with the PPS-based individual pathology models**.

Figure 5 displays the evaluation results of our integrative models for the prediction of 21 pathological findings. We observe that overall prediction performance is quite impressive, increasing sensitivity considerably (approximately 84% on average) without much sacrifice of specificity (approximately 83% on average). In addition, the variability of the prediction performance for all pathologies in the integrative model is much lesser than the individual pathology models it is comprised of, indicating that an integrative approach can provide more stable performance. Figure 4 shows the ROC curves of the integrative models for the 6 pathological findings (3 in liver and 3 in kidney). It is noted that the ROC curves of the IPS-based integrative models are located completely above ROC curves of the PPS-based individual models in several cases. We also measured the area under the ROC curves (AUC) of the IPS-based integrative models and the PPS-based individual models (see Figure 6). As seen in Figure 6, the IPS-based integrative models showed much better performance than the PPS-based individual models overall. On average, approximately 20% of AUC in the proposed integrative models increased over all the pathology prediction, compared to individual pathology models. Particularly, for some pathological findings like liver single cell necrosis and kidney cytoplasmic vacuolization, the IPS-based integrative models achieved distinct improvement in prediction performance. For example, regarding liver single cell necrosis, the IPS-based integrative model can make high quality predictions within 93% of its AUC, but the PPS-based individual models only display this performance within 26% of AUC.

Furthermore, we compared our integrative model performance with the prediction performance given by the recent work of Bowles et al. (2013) [8] which also employs TG-GATEs to train and evaluate the prediction model. Table 2 shows the AUC comparison of the both models in predicting liver toxicity only, since the work of Bowles et al. only concerned itself with liver toxicity. Even if it shows limited comparison in predicting only three liver pathological findings (liver necrosis, hypertrophy, cellular infiltration), our integrative model performance seems to be comparable to their work.

**Table 2:** AUC comparison of the proposed integrative model with the work of Bowles et al.

	Liver necrosis	Liver hypertrophy	Liver cellular infiltration
**Bowles et al.'s model**	0.87	0.97	0.88

**Our integrative model**	0.87	0.91	0.89

## Conclusions

We introduced an integrative model approach for multi-organ drug-induced toxicity prediction. For the development of integrative models, we investigated pathology relationships by considering the co-occurrence of targeted pathological states in training samples, and used them to unite individual pathology prediction models. Consequently, our integrative models produced better prediction performance over all of the 21 targeted pathological findings, than individual pathology prediction models.

Even when the performance of individual pathology prediction models is extremely low, our integrative models could improve the prediction performance considerably by referring to the prediction results of other highly associated pathological findings. In this work, our prediction models were developed using limited training set, so it is expected that more training samples would make the better estimation of the real associations among toxicity pathological findings.

## Competing interests

The authors declare that they have no competing interests.

## Authors' contributions

Jinwoo Kim designed and performed the experiments, and prepared the manuscript. Miyoung Shin advised on detailed methods of the experiments, and corrected the expressions of the manuscript.

## Supplementary Material

Additional file 1 **An example of slide image extracted from the liver and kidney of an in-vivo sample**. Click here for file

Additional file 2 **A table of detected pathological symptoms in each part of the liver and kidney**. This table is formatted for Excel. The table contains type of pathology, part of liver of kidney in which the pathology occurred, and number of drugs which induced the pathology within experiments of TG-GATEs. Click here for file

Additional file 3 **A table of in-vivo samples which were used to evaluate the prediction models**. This table is formatted for Excel. The table contains sample ID, type of medicated drug, and occurrences of drug-induced targeted pathologies for each sample. Click here for file

Additional file 4 **An image to describe the cross-validation method for evaluating toxicity prediction models**. The image shows the concept of cross-validation methods used to evaluate individual pathology prediction models or integrative models. Click here for file

Additional file 5 **ROC curves images for each 21 pathology prediction models**. These images are zip-compressed. Figure 4 is a part of these images. Click here for file

## Declarations

Funding for the publication of the article was provided by the Ministry of Food and Drug Safety, Korea.

This article has been published as part of *BMC Bioinformatics *Volume 15 Supplement 16, 2014: Thirteenth International Conference on Bioinformatics (InCoB2014): Bioinformatics. The full contents of the supplement are available online at http://www.biomedcentral.com/bmcbioinformatics/supplements/15/S16.
