# Supplementary figures and images for: An integrative model of multi-organ drug-induced toxicity prediction using gene-expression data
Source: BMC Bioinformatics. 2014 Dec 8;15(Suppl 16):S2. doi: 10.1186/1471-2105-15-S16-S2 (PMC4290650; doi:10.1186/1471-2105-15-S16-S2)

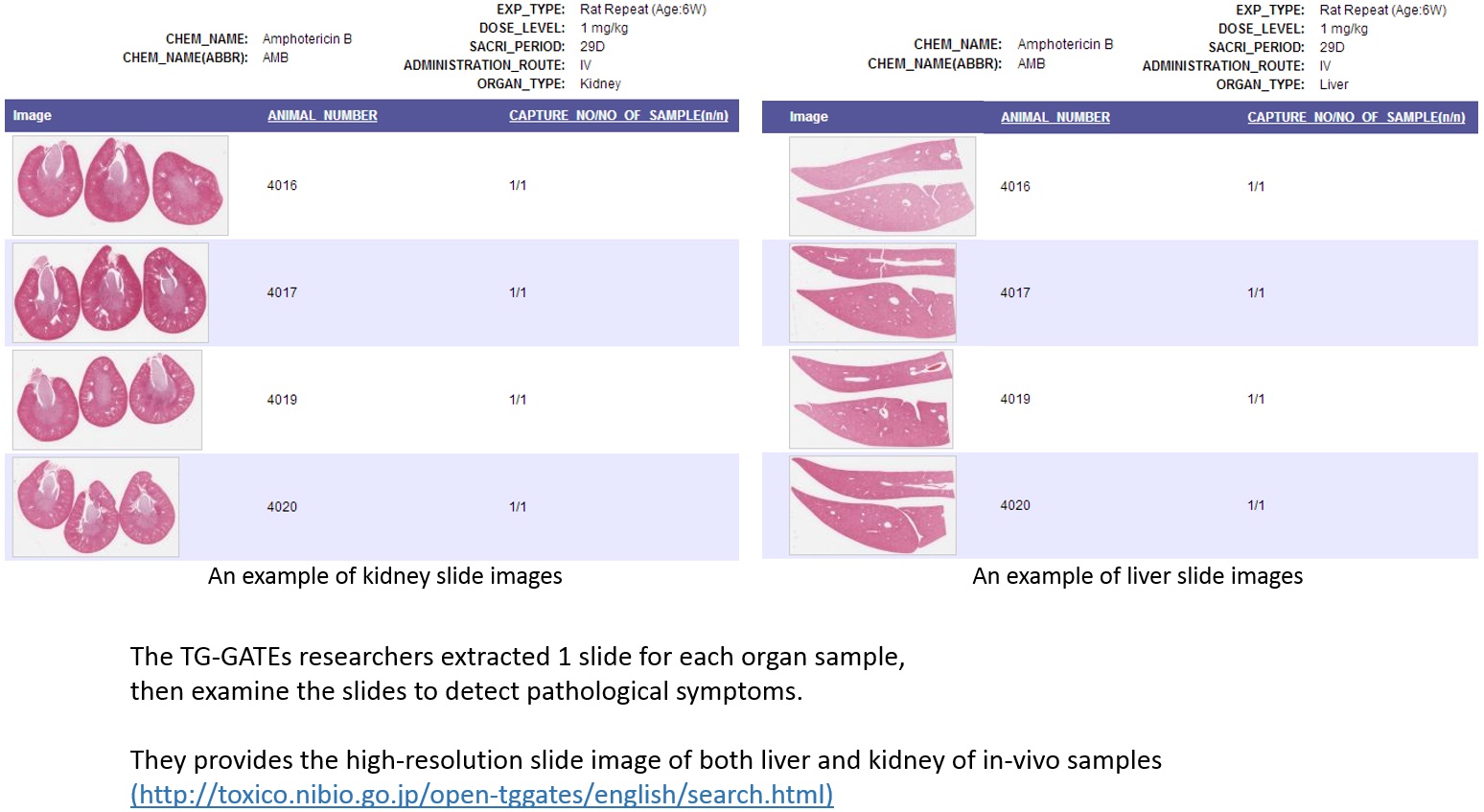

Supplement: Additional file 1 — An example of slide image extracted from the liver and kidney of an in-vivo sample. [file 1471-2105-15-S16-S2-S1.jpg]

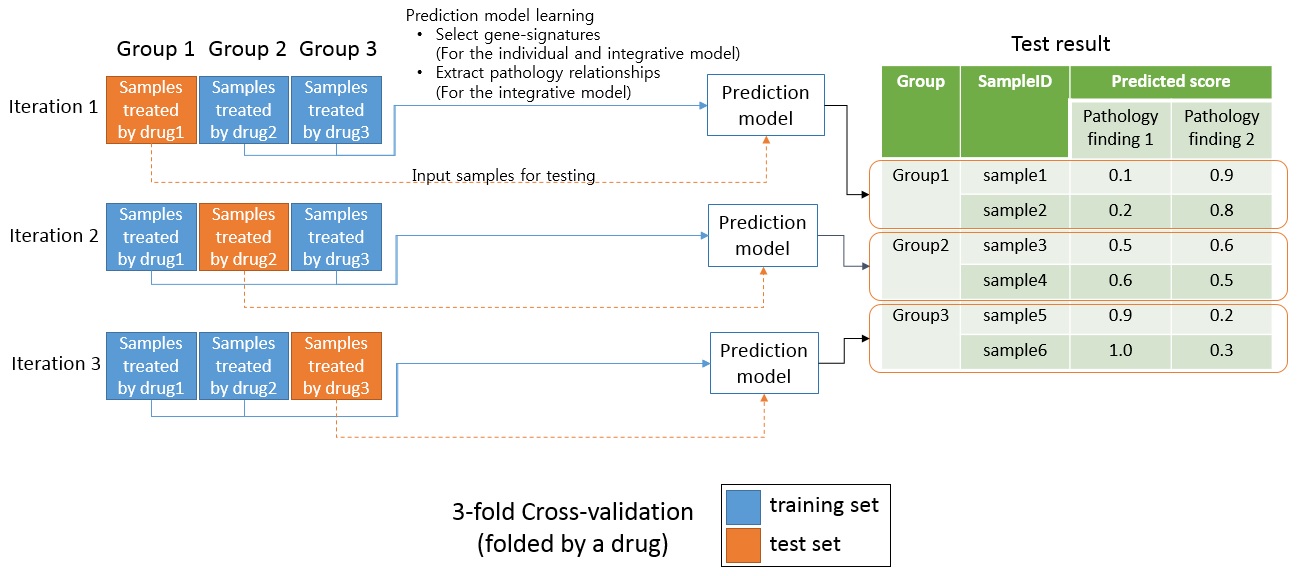

Supplement: Additional file 4 — An image to describe the cross-validation method for evaluating toxicity prediction models. The image shows the concept of cross-validation methods used to evaluate individual pathology prediction models or integrative models. [file 1471-2105-15-S16-S2-S4.jpg]

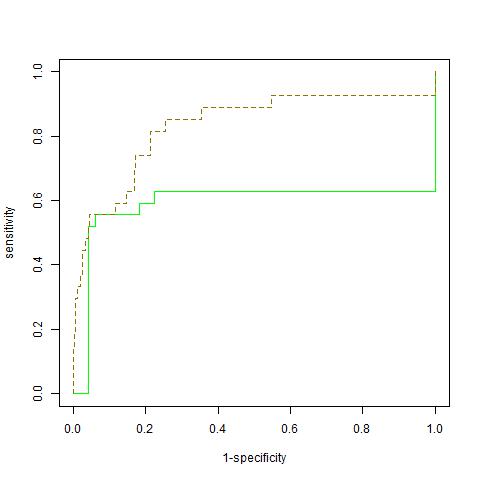

Supplement: Additional file 5 — ROC curves images for each 21 pathology prediction models. These images are zip-compressed. Figure 4 is a part of these images. [file 1471-2105-15-S16-S2-S5.zip › Kidney Vacuolization, cytoplasmic.jpg]

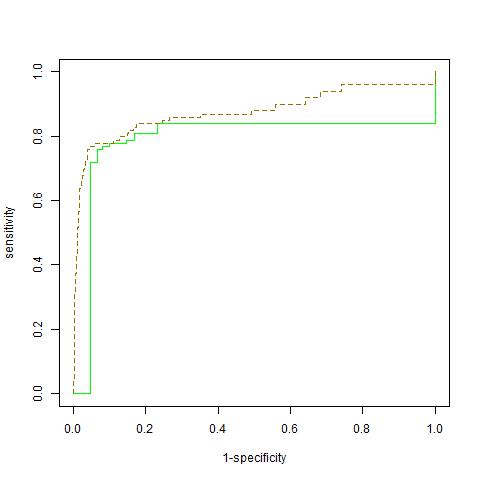

Supplement: Additional file 5 — ROC curves images for each 21 pathology prediction models. These images are zip-compressed. Figure 4 is a part of these images. [file 1471-2105-15-S16-S2-S5.zip › Kidney Regeneration.jpg]

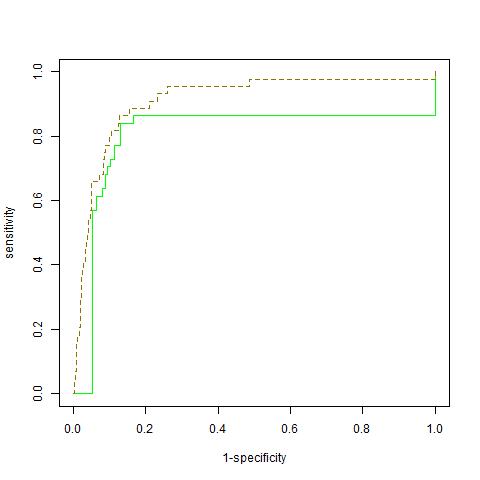

Supplement: Additional file 5 — ROC curves images for each 21 pathology prediction models. These images are zip-compressed. Figure 4 is a part of these images. [file 1471-2105-15-S16-S2-S5.zip › Kidney Change, basophilic.jpg]

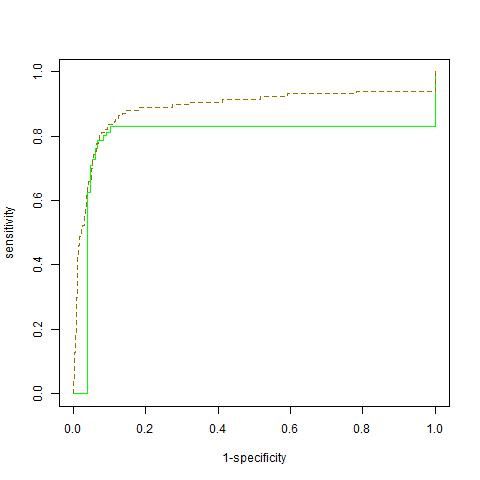

Supplement: Additional file 5 — ROC curves images for each 21 pathology prediction models. These images are zip-compressed. Figure 4 is a part of these images. [file 1471-2105-15-S16-S2-S5.zip › Kidney Dilatation.jpg]

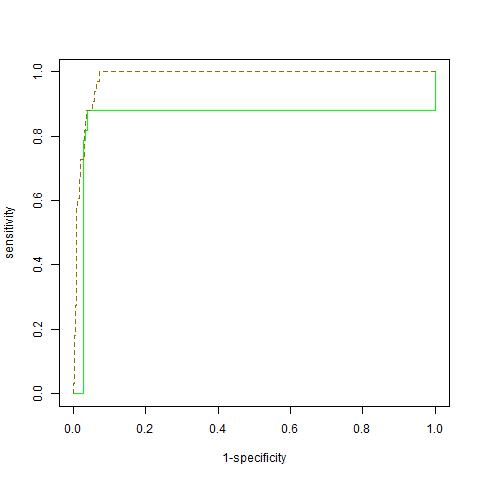

Supplement: Additional file 5 — ROC curves images for each 21 pathology prediction models. These images are zip-compressed. Figure 4 is a part of these images. [file 1471-2105-15-S16-S2-S5.zip › Kidney Cast, hyaline.jpg]

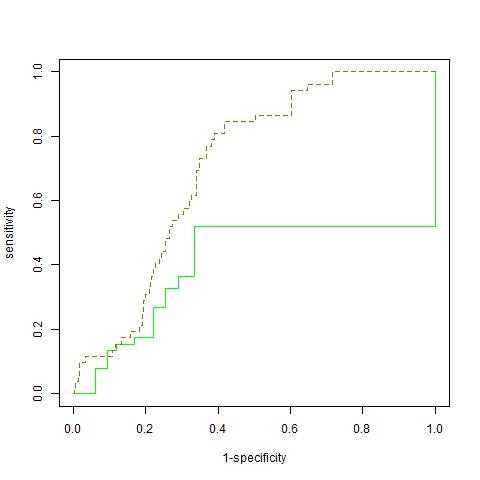

Supplement: Additional file 5 — ROC curves images for each 21 pathology prediction models. These images are zip-compressed. Figure 4 is a part of these images. [file 1471-2105-15-S16-S2-S5.zip › Kidney Hyaline droplet.jpg]

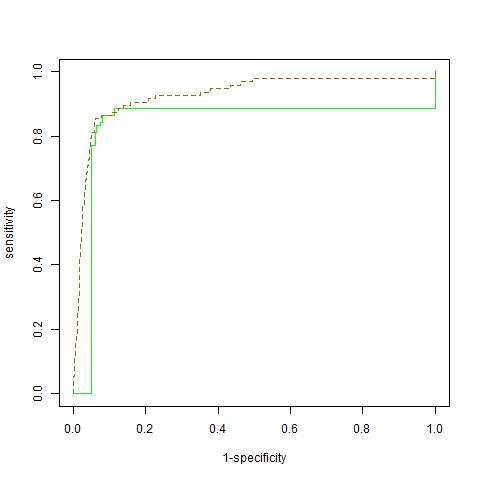

Supplement: Additional file 5 — ROC curves images for each 21 pathology prediction models. These images are zip-compressed. Figure 4 is a part of these images. [file 1471-2105-15-S16-S2-S5.zip › Kidney Necrosis.jpg]

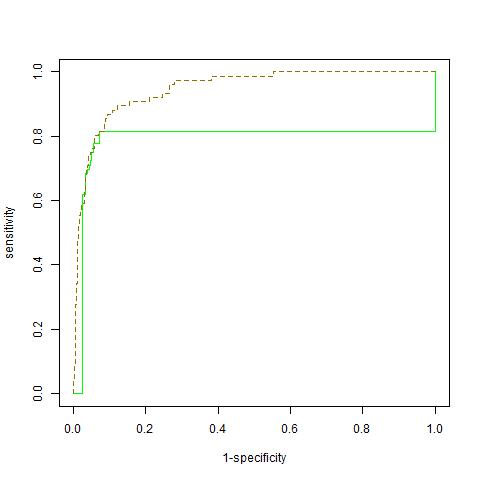

Supplement: Additional file 5 — ROC curves images for each 21 pathology prediction models. These images are zip-compressed. Figure 4 is a part of these images. [file 1471-2105-15-S16-S2-S5.zip › Kidney Degeneration.jpg]

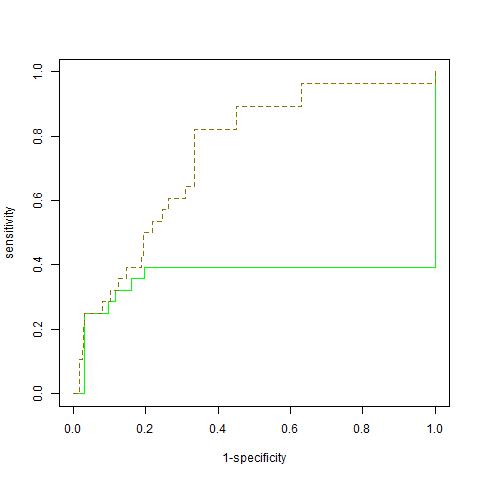

Supplement: Additional file 5 — ROC curves images for each 21 pathology prediction models. These images are zip-compressed. Figure 4 is a part of these images. [file 1471-2105-15-S16-S2-S5.zip › Kidney Hypertrophy.jpg]

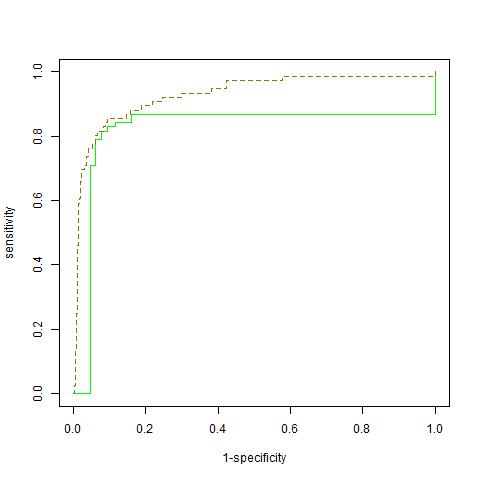

Supplement: Additional file 5 — ROC curves images for each 21 pathology prediction models. These images are zip-compressed. Figure 4 is a part of these images. [file 1471-2105-15-S16-S2-S5.zip › Kidney Cellular infiltration.jpg]

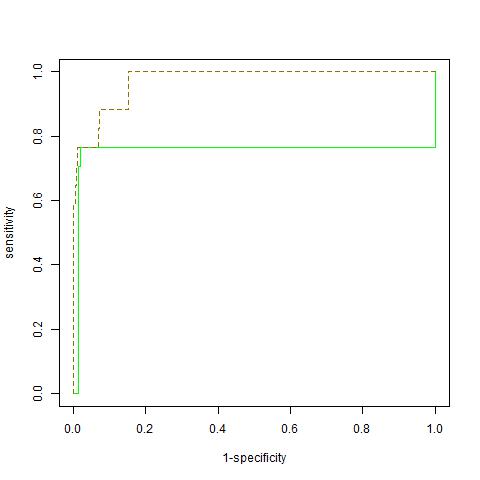

Supplement: Additional file 5 — ROC curves images for each 21 pathology prediction models. These images are zip-compressed. Figure 4 is a part of these images. [file 1471-2105-15-S16-S2-S5.zip › Liver Fibrosis.jpg]

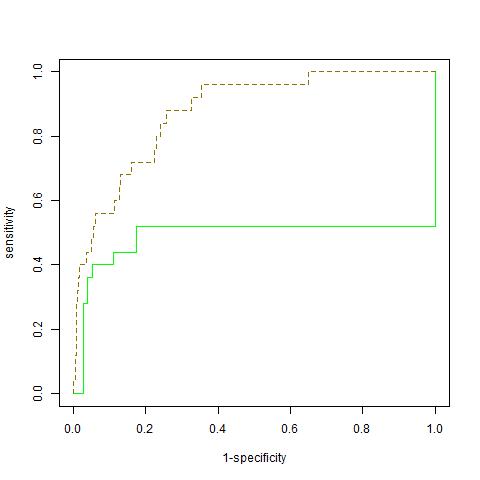

Supplement: Additional file 5 — ROC curves images for each 21 pathology prediction models. These images are zip-compressed. Figure 4 is a part of these images. [file 1471-2105-15-S16-S2-S5.zip › Liver Proliferation.jpg]

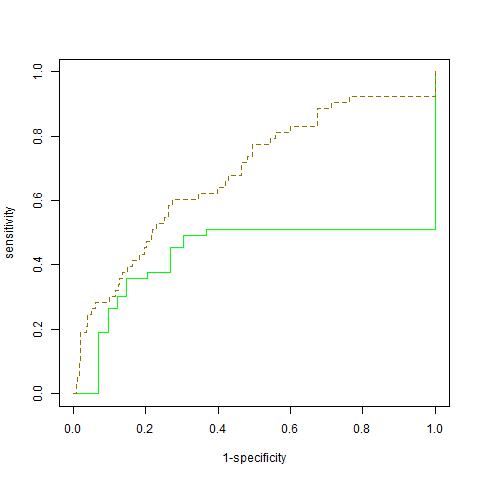

Supplement: Additional file 5 — ROC curves images for each 21 pathology prediction models. These images are zip-compressed. Figure 4 is a part of these images. [file 1471-2105-15-S16-S2-S5.zip › Liver Vacuolization, cytoplasmic.jpg]

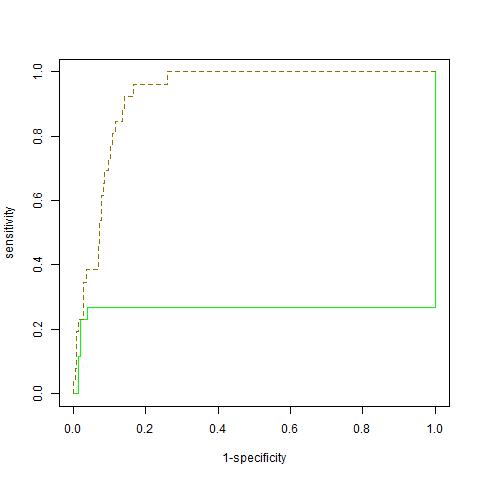

Supplement: Additional file 5 — ROC curves images for each 21 pathology prediction models. These images are zip-compressed. Figure 4 is a part of these images. [file 1471-2105-15-S16-S2-S5.zip › Liver Single cell necrosis.jpg]

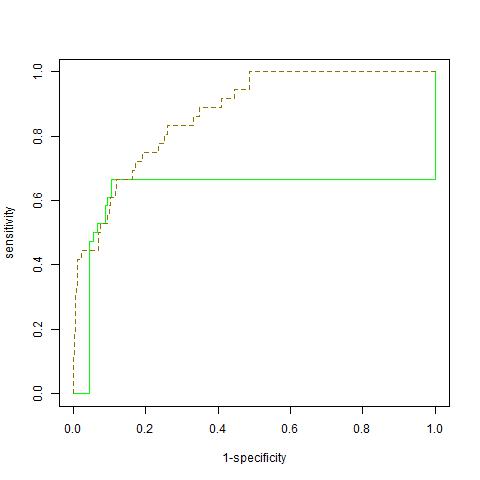

Supplement: Additional file 5 — ROC curves images for each 21 pathology prediction models. These images are zip-compressed. Figure 4 is a part of these images. [file 1471-2105-15-S16-S2-S5.zip › Liver Necrosis.jpg]

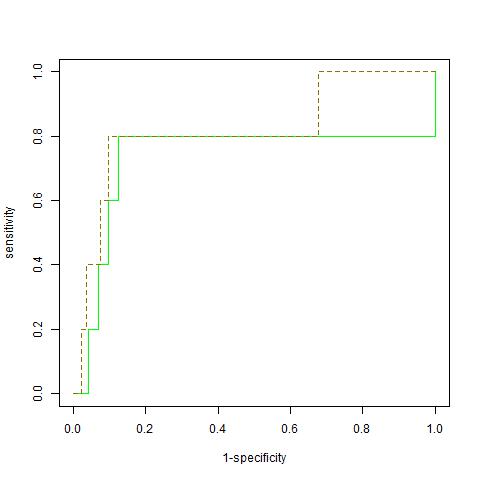

Supplement: Additional file 5 — ROC curves images for each 21 pathology prediction models. These images are zip-compressed. Figure 4 is a part of these images. [file 1471-2105-15-S16-S2-S5.zip › Liver Deposit, pigment.jpg]

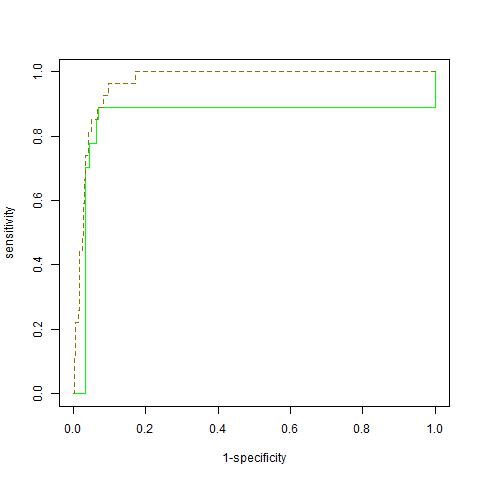

Supplement: Additional file 5 — ROC curves images for each 21 pathology prediction models. These images are zip-compressed. Figure 4 is a part of these images. [file 1471-2105-15-S16-S2-S5.zip › Liver Increased mitosis.jpg]

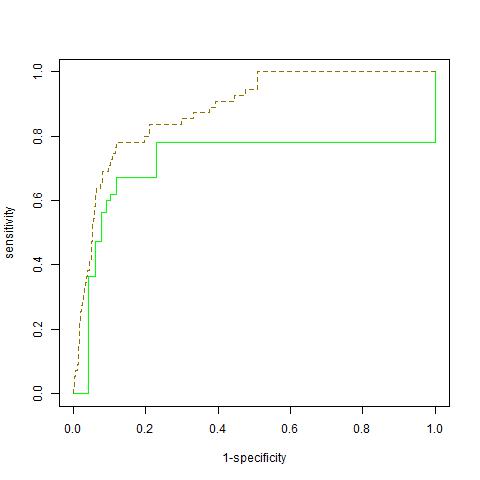

Supplement: Additional file 5 — ROC curves images for each 21 pathology prediction models. These images are zip-compressed. Figure 4 is a part of these images. [file 1471-2105-15-S16-S2-S5.zip › Liver Degeneration.jpg]

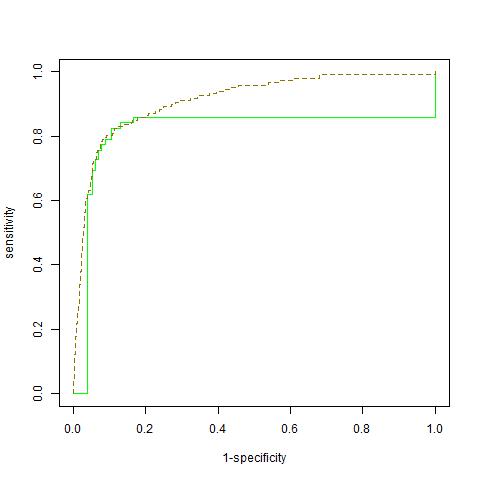

Supplement: Additional file 5 — ROC curves images for each 21 pathology prediction models. These images are zip-compressed. Figure 4 is a part of these images. [file 1471-2105-15-S16-S2-S5.zip › Liver Hypertrophy.jpg]

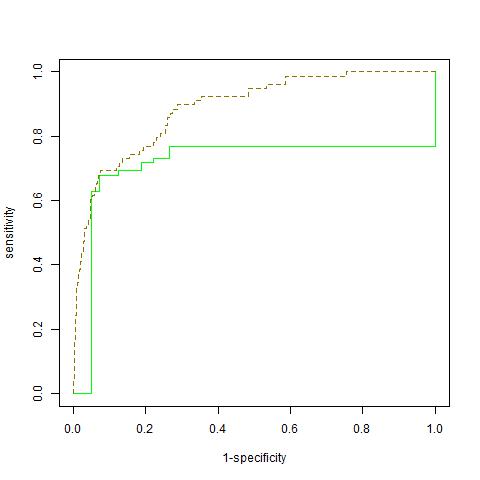

Supplement: Additional file 5 — ROC curves images for each 21 pathology prediction models. These images are zip-compressed. Figure 4 is a part of these images. [file 1471-2105-15-S16-S2-S5.zip › Liver Cellular infiltration.jpg]

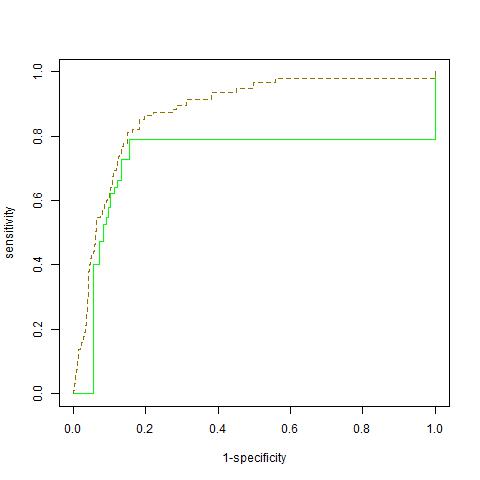

Supplement: Additional file 5 — ROC curves images for each 21 pathology prediction models. These images are zip-compressed. Figure 4 is a part of these images. [file 1471-2105-15-S16-S2-S5.zip › Liver Change, eosinophilic.jpg]
